# Method and Computer System for Dialog Optimization of Aging Biomarker Panels for Biological Age Assessment

**DOI:** 10.3389/fgene.2021.634734

**Published:** 2021-03-05

**Authors:** Vyacheslav N. Krut'ko, Vitaly I. Dontsov, Nina A. Ermakova, Valentina V. Makarova, Oleg V. Mitrokhin, Ekaterina A. Shashina, Denis V. Shcherbakov

**Affiliations:** ^1^Institute for Systems Analysis Federal Research Center “Computer Science and Control” of Russian Academy of Sciences, Moscow, Russia; ^2^Sechenov First Moscow State Medical University, Moscow, Russia

**Keywords:** aging, diagnostics of aging, biological age, biomarkers of aging, dialog optimization, personalized medicine

## Abstract

A concept, method, algorithm, and computer system (CS) of step-by-step dialog optimization of biomarker (BM) panels for assessing human biological age (BA) according to a number of universal criteria based on incomplete and noisy data have been developed. This system provides the ability to automatically build BM panels for BA assessment and to increase the accuracy of BA determination while reducing the number of measured BMs. The optimization criteria are as follows: high correlation of BMs with chronological age (CA); minimum size of BM panels, obtained by rejecting highly cross-correlated BMs; high accuracy of BA assessment; high accuracy of BA/CA dependency interpolation; absence of outliers in BM values, which reduce the BA assessment accuracy; rejection of panels resulting in a high standard deviation for the BA-CA difference; and possible additional criteria entered by the researcher according to the task specifics. The CS input consists of data on physiological, biochemical, and other BMs that change with age. The CS output is a panel of BMs optimized according to the specified optimization criteria. The CS is user-friendly. It allows the user to add optimization criteria that the researcher considers to be important or to remove criteria that the user considers incorrect. The CS may be used in solving practical problems of anti-aging medicine, such as the treatment and prevention of age-related chronic non-infectious diseases representing the main causes of death. The authors' point of view on the role and place of BA diagnostics in this area is discussed.

## Introduction

The global problem of population aging has significantly increased research interest in both the mechanisms of aging and the search for methods for decelerating and reversing aging (Moskalev et al., 2016, 2017; Vaiserman and Lushchak, 2017; Krut'ko et al., 2018). The fundamental basis of these studies is the methods for quantitative assessment of aging levels both of the body as a whole and of its individual vital organs and systems; in other words, methods for assessing the biological age (BA) of the body as a whole and the partial biological age (BAp) of its vital systems (Dean, 1988; Mooradian, 1990; Balin, 1996; McClean, 1997; Anstey and Smith, 1999; Krøll and Saxtrup, 2000; DeCarlo et al., 2014; Negasheva et al., 2014; Dontsov and Krut'ko, 2015; Moskalev et al., 2016, 2017; Finkel et al., 2017; Mitnitski and Rockwood, 2019). Moreover, while there are many different methods for BA assessment in literature, there is still no answer to the question: “Which of these methods is the best?” This paper contains an attempt to answer this question.

The purpose of our article is to try to solve several theoretical and practical problems of interest to gerontology, namely, (1) to give one of the possible answers to the key question of Ward Dean (Dean, 1988), which is presently relevant, “It remains unclear which of the panels presented is the best and can be recommended for wide practical use”; (2) to offer, in our opinion, a reasonable criterion for selection of the best panels of aging biomarkers (BMs) for specific research tasks; and (3) to create a computer system (CS) that facilitates the application of this criterion for solving various practical problems of creating optimal (i.e., accurate and easy-to-use) panels of aging BMs for BA assessing.

Different views on the concepts of “biological age” and “aging” are presented in literature and may exist. We adhere to the following views:

- a person ages with years, and it is expressed as a decrease in bodily functions;
- there are average age norms for these functions;
- comparison of function values of a particular person with age norms determines the BAp of these functions;
- and the weighted sum of the BAp of the vital functions of the organism determines the BA of the organism as a whole.

## Methods and Materials

The standard input data loaded into the CS is an N × M rectangular Excel spreadsheet containing data on N values of BMs for M clients [the set of BMs included the calendar (chronological) age (CA) of the clients]. However, it is not necessary to have all BM data for each client. An example of the practical CS application is based on spreadsheets of size 15 × 160 (for women) and 15 × 33 (for men), containing data for a group of clients examined at the Russian National Gerontological Center (www.ngcrussia.org/) to obtain recommendations for individual anti-aging programs.

These data were processed using the following algorithms:

- calculation of correlations of BMs with CA,
- calculation of BMs cross-correlations,
- calculation of mean values and standard deviations for BMs and BAp in the examined panel for separate BMs and deviations of BA from CA,
- consideration of measurement accuracy and BM age range,
- exclusion of maximum and minimum values of BMs within age ranges.

As a result, an optimized BAp was calculated for each BM.

## Results

The method was implemented using the CS, which was developed on Object Pascal software with Delphi 7 application development system. The CS provides step-by-step dialog optimization of BM panels and thereby assists in obtaining formulas for BA and BAp calculation for the optimized panels and the results of calculations by these formulas.

The optimization criteria for this CS are as follows:

- high correlation of BMs with CA,
- minimum size of BM panels, obtained by rejecting highly cross-correlated BMs,
- high accuracy of BA assessment,
- high accuracy of interpolation of BA/CA dependency,
- absence of outliers in BM values, which reduce the BA assessment accuracy,
- rejection of panels resulting in a high standard deviation for the BA-CA difference,
- possible additional criteria entered by the researcher according to the task specifics.

The CS is user-friendly. It allows the user to add optimization criteria that the researcher considers important or remove criteria that he or she considers incorrect (for example, discussion criteria related to CA).

The CS operational algorithm is as follows:

- *At the first step* the correlations of BMs with CA are determined, and BMs with low correlation are rejected. The rejection threshold may be pre-specified in the CS or else determined by the researcher in the dialog mode.
- *At the second step* the BM sets are checked for redundancy among the BMs selected for the same panel using the calculated BM cross-correlations.
- *At the third step* the accuracy of each BM assessment is determined based on the instrument accuracy of the BM measurement procedure, the range of interindividual BM assessment fluctuations in the group studied, the magnitude of BM variation in the reference age interval, and the size of the age interval for which the BM is assessed.
- *At the fourth step* the BM/CA graphs are plotted, and the linear, exponential, and polynomial regression formulas for each BM, as well as the inverse formula of BA determination for a given BM value, are calculated. To correct the graphs, the extreme BM values are rejected.
- *At the fifth step* a BA/CA graph is plotted (a typical BA graph will, in theory, show little deviation of values from the diagonal of the BA/CA square), and a standard deviation σ for the difference (BA-CA) is calculated. In the group of clients examined, there are typically individuals with both higher and lower BA compared to their CA. In our experience, σ <10 years is an acceptable value.
- *At the sixth step* additional BM criteria, determined by the specifics of the goals and objectives of the BA study or the practical CS application, may be introduced: for example, the possibility of sharp BM deviations from expected age standards due to sports training or illness may be taken into account.
- *At the seventh step* the selected BMs are used to create panels to determine BA and BAp according to the specifics of the goals and objectives of the BA study or the practical CS application.

**Figure 1:**
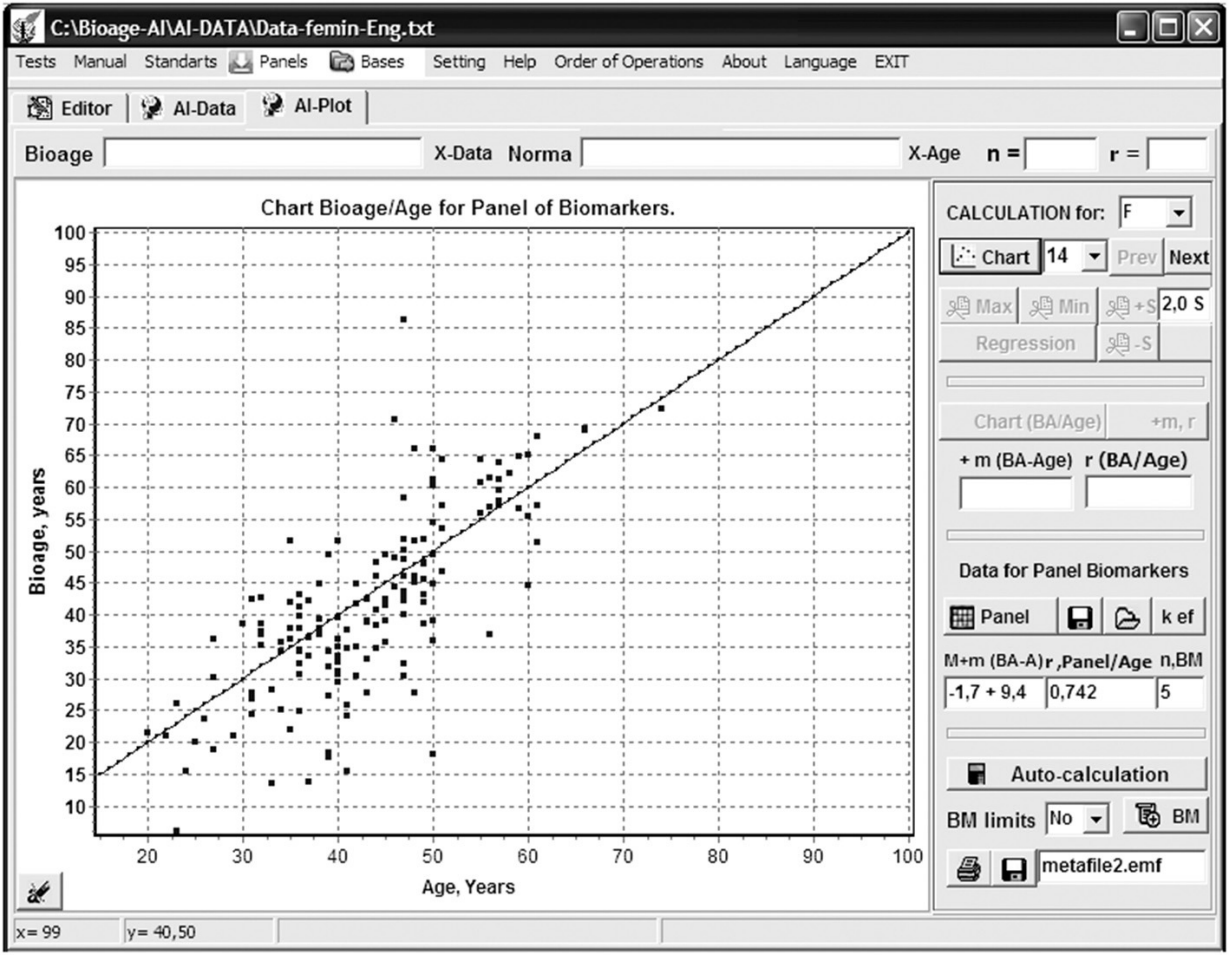
Program “Constructor of aging biomarkers panels”: a dialog panel of setting parameters for biomarkers selection and bioage calculation.

The above processes are carried out in dialog mode using the CS dialog box (Figure 1). In this mode, the BM selection criteria, determined by the researcher, are introduced: the rejection threshold for BMs demonstrating a low level of correlation with CA; the threshold for BM cross-correlation (when exceeded, a BM may be rejected as superfluous); the BM measurement accuracy, which determines the accuracy of the estimate of the corresponding BAp (in years); thresholds for deviations from mean values (the number of sigmas for each 10-year age period, entered separately), so that exceeding maximum and minimum BM values will be rejected as unrepresentative; the number of maximum and minimum BA values that can be rejected; the precision (i.e., number of decimal places) of the coefficients found in the formulas for BA calculation; and the limits for correlation values of BA with CA for the created panels. Moreover, the significance factors (weight factors *w*) of each BM (or corresponding BAp) contribution to the resulting BA may be calculated and taken into account for the *w* values in the range (0, 1). The final BA for the created BM panel is calculated as a weighted mean BAp value for the selected BM set.

The above criteria and limits may be specified before commencement of the CS operation, and then all optimization steps will be carried out by the CS automatically, without human intervention. These criteria and limits may also be introduced at each step, if the researcher wants to consider the results of calculations obtained at previous steps of CS operation, e.g., the significance and accuracy of individual BM assessments.

### Example of Computer System Operation

An example of the CS operation is given below. The objective of the study was to determine the BM panel resulting in a maximum correlation of BA with CA while using a minimum number of selected BMs and producing a minimum difference value (BA-CA).

Data for two groups of clients (33 men and 160 women) were examined separately using a set of BM values included in the panel officially approved in the USSR for BA assessment (Vojtenko et al., 1984). This panel includes the following BM:

APs, APd and APp – systolic, diastolic and pulse arterial pressure (mmHg),
PWVe: pulse wave velocity through the artery of the elastic type (m/sec),
PWVm: pulse wave velocity through the artery of the muscular type (m/sec),
LC: lung capacity (ml),
BHT: breath hold time as you exhale (sec),
A: eye accommodation (distance to the closest point of clear vision, expressed in diopters),
HA: hearing acuity or hearing threshold at 4,000 Hz (dB),
SB: static balancing (sec) on the left foot,
BW: body weight (kg),
SAH: self-assessment of health test (scores),
WT: Wechsler's test (scores).

At the stage of assessing the correlations of BMs with CA, extremely low correlations with age were noted for BHT (*r* = −0.047) and SAH (*r* = 0.232); moreover, there were high cross-correlations for the following parameters: APs/APd (*r* = 0.795), APs/APp (*r* = 0.803), PWVe/PWVm (*r* = 0.986), and LC/BW (*r* = 0.657). This provides the need of excluding two of three factors (APd and APp) of blood pressure assessment from the BA assessing panels, as well as one of two parameters (PWVe or PWVm) of artery elasticity assessment. What specific factors should be excluded may be determined by considering their assessment accuracy.

The accuracy of BAp assessment (in years) depends on the accuracy of the BM measurement method, the magnitude of BM change in the measured age interval, the size of this age interval, and the size of interindividual differences of BM in the studied group. There are two classes of the most common and important BA and BAp assessment tasks.

The first class includes tasks of longitudinal assessment of BA and BAp changes in individuals under the influence of personal aging prevention programs. In this case, there are no interindividual differences, and the accuracy of assessment depends on the ratio of BM measurement method accuracy to the BM changes within the reference research range, usually having a value from several months to several years. For example, for APs changing from 120 to 160 mmHg (change = 40 mmHg) in the age range 20–70 years (range = 50 years) with a hardware measurement accuracy 5 mmHg, the calculated accuracy value = 40 mmHg/5 mmHg = 8 units for 50 years, or 50 years/8 registered units = 6.2 years represents the accuracy achievable in determining BAp in years. This is marginally acceptable for BAp, but considering the importance of the parameter and the comparison of its value with an earlier one for the same patient (with no interindividual variation), its application is acceptable. The accuracy of BA assessment within ±5 years is considered to be sufficient for this kind of research. Similarly calculated accuracy for APp equals ±12.5 years, which excludes this parameter from the list of parameters acceptable for BA assessment.

The second class includes tasks of population cross-sectional BA studies. For these tasks, it is necessary to consider the values of interindividual dispersion, which almost always significantly exceeds the error of the measurement method. For example, LC variation within the 50-year age interval from 20 to 70 years reaches a 2-fold value with method accuracy <2–3% from the measured value, but interindividual variation also becomes 2-fold. However, the LC parameter is almost always included in the BA assessment panel, since the high measurement accuracy and the large age-dependent variation allow us to obtain sufficiently accurate and reliable data in population studies.

The BMs with the highest instrumental measurement accuracy, smallest interindividual dispersion, and most evident age-related changes are most acceptable for both classes of tasks. For example, PWV is such a BM. An approximate value of PWVe alteration in the 50-year age interval from 20 to 70 years equals 700 m/s (1,200 m/s−500 m/s) with an instrumental measurement accuracy of about 10 m/s and a small interindividual dispersion; BAp for this BM may be determined with an accuracy of 0.8 years and a correlation value with CA of *r* = 0.823.

When the graphs of BW to BM were plotted, it was clearly seen that the possibility of sharp and rapid fluctuations of this parameter throughout life and the significant interindividual dispersion (from 45 to 135 kg in the considered group) prohibits using this BM in the panel for individual BA assessment.

After a similar step-by-step analysis was carried out for all 15 BMs, it was found that the optimal BM panel contains five BMs for women (APs, PWVe, LC, A, WT) and six BMs for men (APs, PWVe, LC, A, BHT, SB). The formulas for BA-CA difference calculated for these panels are as follows:

For women:

BA-CA =

(0.83 × [−44.384 + 0.1235 × PWVe – CA] +

0.65 × [−7.0318 + 0.333 × A – CA] +

0.58 × [−118.760 +1.328 × APs – CA] +

0.54 × [204.880 – 2.8573 × WT – CA] +

0.54 × [226.860 – 0.0662 × LC – CA])/5

For men:

BA-CA =

(0.77 × [−50.098 +0.1325 × PWVe – CA] +

0.53 × [67.658 – 0.632 × SB – CA] +

0.43 × [−38.018 + 0.426 × A – CA] +

0.42 × [319.980 – 0.073 × LC – CA] +

0.38 × [−382.410 + 3.3884 × APd – CA] +

0.38 × [−110.290 + 3.837 × BHT – CA])/6

**Figure 2:**
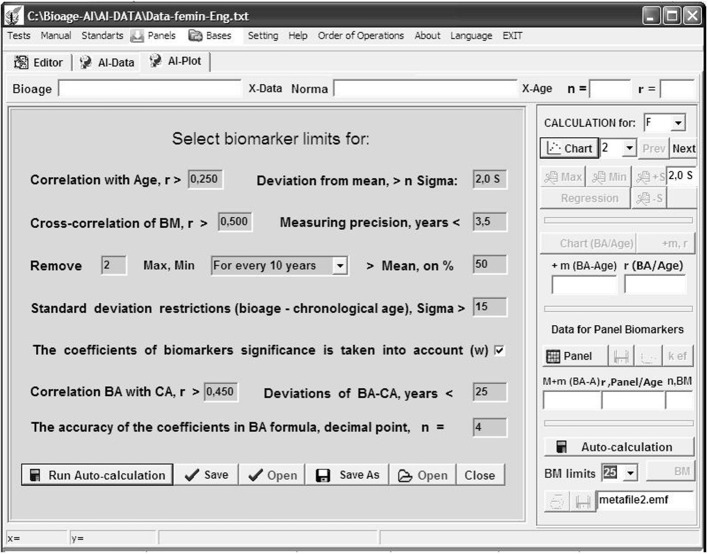
Program “Constructor of aging biomarkers panels”: curve Bioage—Chronological age (Age) for women based on five selected biomarkers.

The accuracy in determining the BA-CA difference was M ± σ = −1.7 ± 9.4 years, with a correlation coefficient *r* = 0.741 for women (Figure 2) and M ± σ = −3.8 ± 7.7 years, *r* = 0.792 for men.

For comparison, the official BM panel of the USSR, including 15 BMs, had an accuracy for women M ± σ = −4.1 ± 28.2 years, *r* = 0.324.

The accuracy may still be improved by excluding the maximum and minimum outlier BM values and entering weight factors of the significance of BMs (*w*) into the formulas, which are expertly evaluated based on the functional significance of the processes and mechanisms referred to by those BMs for the aging processes of the whole body, as well as both the researcher's capabilities and the client's wishes regarding the set of BMs they prefer to use for one or another reason.

All stages of calculations may be carried out automatically or step-by-step in dialog mode. As a result, optimal (according to the above criteria) BMs are selected, which may be saved in the CS database for further use: creating effective new panels or adding to existing panels for BA assessment of individual clients.

The developed CS of step-by-step dialog optimization of BMs for the assessment of BA may work with an unlimited set of data based on incomplete and noisy data and according to the requirements and preferences of the researcher.

## Discussion

Currently, there is a problem of different perspectives in the literature regarding the concept of “biological age” and the quality criteria of various BMs of aging. Our work addresses the practical issues. The general consideration of this problem is beyond the scope of this article.

The first serious generalization of the results of solving the problem of creating panels of BMs of aging for human BA assessing was given in Ward Dean's book *Biological Aging Measurement* (Dean, 1988). The book described about two dozen BM panels and the corresponding formulas for determining BA, which were created by teams of scientists from different countries and which contained from a few to several dozen BMs. After describing all these panels in detail, Ward Dean ended his book with an at-the-time unsolved question: “It remains unclear which of the presented panels is the best and can be recommended for wide practical use.”

From the control science it is well-known that the task of finding the optimal solution is correctly set if an optimality criterion or a group of criteria is specified. After the publication of Ward Dean's book, many authors tried to make the best BM panels for BA determination, offering their own optimality criteria (Dean, 1988; Mooradian, 1990; Balin, 1996; McClean, 1997; Anstey and Smith, 1999; Krøll and Saxtrup, 2000; DeCarlo et al., 2014; Negasheva et al., 2014; Dontsov and Krut'ko, 2015; Moskalev et al., 2016, 2017; Finkel et al., 2017).

The conceptual view of the authors of this paper on the problem of BM panels creating for BA assessing, with the resulting method that implements this view, is that posing the problem in terms of finding a single optimal (best) panel is, in general, incorrect, since there are many scientific and practical problems in gerontology and anti-aging medicine, each of which involves its own system of optimality criteria reflecting the specifics of those tasks and the BM sets corresponding to these tasks—in particular, diverse physiological, functional, genetic, epigenetic, and other BMs.

However, among the many criteria, there are several fairly obvious adaptive optimality criteria suitable for any panel and serving to improve the accuracy and ease of use of these panels. In this regard, the authors conceived and implemented the idea (presented in this paper) of creating a tool that automatically takes these criteria into account when developing optimal BM panels for BA assessment, which greatly facilitates the work of a researcher or practitioner working in the field of anti-aging medicine or treatment or prevention of age-related chronic non-communicable diseases (CNCD) that are the main causes of death.

The CS offers a gentle and user-friendly interface. Any optimization criteria may be excluded during the dialogue; additional criteria reflecting the specifics of the user's tasks (e.g., the availability of this or that equipment for BM diagnostics, the cost of BM assessment, time constraints for the whole BA assessment procedure, and others) may be introduced, and the fully automatic operation mode, convenient for servicing a large flow of clients, may be used.

The use of the CS for the optimization of the BM panel officially approved in the USSR (Vojtenko et al., 1984) allowed 3-fold reduction of the number of BMs, increased the accuracy of BA determination, and permitted the use of a noisy and incomplete BM sampling to calculate BA.

Fundamental degenerative processes of aging are the basis of CNCD—the main cause of death (Blumenthal, 2003; Kipling et al., 2004; Marengoni et al., 2011). These processes may be combined into several aging syndromes (Krut'ko et al., 2018), each of which usually corresponds to one or more CNCD syndromes. In particular, the following main aging syndromes may be selected: tissue sclerosis syndrome, tissue hypoxia syndrome, intoxication syndrome, oxidative stress syndrome, immune deficiency syndrome, maladaptation syndrome, physical senility syndrome, metabolic disorder syndrome, hormonal disorder syndrome, social isolation of elderly persons and psychological age-related changes.

All of these syndromes may be associated with groups of specific BMs characterizing the level of age-related degradation of the body system under consideration and determining the BAp of this system. For example, the following set of BMs may be applied to tissue sclerosis syndrome: pulse wave velocity, systolic arterial pressure, blood oxygen saturation, lung capacity.

In each specific case this set of BMs may be determined by a dedicated physician; naturally, he or she is faced with the task of determining the desired BAp using an optimal method, i.e., in the most accurate and simplest way, without requiring special knowledge of mathematics and computer technology. In this case our CS may be useful. In our opinion, in this case the diagnosis of current BA or BAp is not of the greatest interest, but the determination of the individual changes (ΔBA and ΔBAp) in specific clients subject to personalized anti-aging programs or undergoing the prevention and treatment of CNCD is very interesting.

The initial assessment of BA and BAp is of course not very accurate, as it is determined on the basis of the reference group data where BMs have sufficiently large inter-individual dispersion. The assessment of changes—ΔBA and ΔBAp—is protected from significant errors due to interindividual dispersion depending on individual BM changes during the implementation of anti-aging programs. These changes are much more informative because they show the effect of prevention or treatment programs and indeed the fine structure of this effect, represented by the pattern of changes in specific BMs included in the panel. Another useful task that can be solved here is the use of the “general checkup” method—a comprehensive BAp assessment of the main vital systems of the body, which makes it possible to reveal the most rapidly aging system—a weak link that accelerates the body's approach to death.

Finally, it is interesting to note that the logic of the CS operation is similar to that of deep machine learning of a neural network, where the steps of the CS correspond to the layers of that network and more and more aggregation of the processed information about the object takes place at each step. This opens a way for further CS improvement by adding artificial intelligence and voice assistance to its functions, provided there is a sufficiently large BM database (today these data are rapidly accumulating all over the world) to help the researcher become more effective in solving the problems of aging and risk management for CNCD. Such approaches are currently effectively used to solve BA assessment problems (Putin et al., 2016; Vidaki et al., 2017).

## Data Availability Statement

The original contributions presented in the study are included in the article/supplementary material, further inquiries can be directed to the corresponding author.

## Author Contributions

VK: concept and selection of optimization criteria for biomarker panels. VD: concept and algorithms of computer system realization. NE: preparation of experimental data. VM: the choice of biomarkers and interpretation of the program. OM: examples and scenarios of using a computer system. ES: help with writing an article and assistance in the translation of the article. DS: computer system programming. All authors contributed to the article and approved the submitted version.

## Conflict of Interest

The authors declare that the research was conducted in the absence of any commercial or financial relationships that could be construed as a potential conflict of interest.

## Abbreviations

CS: computer system
CA: chronological age
BM: biomarker
BA: biological age
Bap: partial biological age (biological age of separate body system).
